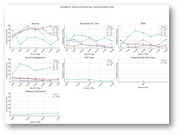# Unveiling the Everyday: Ethnographic observation of persons living with dementia in a long‐term care facility in Dubai, United Arab Emirates

**DOI:** 10.1002/alz70858_104770

**Published:** 2025-12-26

**Authors:** Seada A. Kassie, Arlene Astell

**Affiliations:** ^1^ University of Reading, Reading, Bulmershe Court, United Kingdom; ^2^ Middlesex University Dubai, Dubai, Dubai, United Arab Emirates; ^3^ Northumbria University, Newcastle upon Tyne, United Kingdom; ^4^ University of Toronto, Toronto, ON, Canada; ^5^ University of Reading, Reading, United Kingdom

## Abstract

**Background:**

The behaviours of four residents living with dementia was analysed using ethnographic observation techniques while they were receiving routine care in a long‐term care facility in Dubai, United Arab Emirates (UAE). The aim was to investigate if dementia care staff in Dubai are equipped with the right skills and resources to provide person‐centred dementia care to people living with dementia.

**Method:**

The residents were observed during their waking hours from 7:00 to 21:00, for a time‐block of five minutes, followed by 10 minutes for note calibration, and another five minutes used for breaks before resuming observation for another block of five minutes. In total, 840 to 870 minutes of data from each resident was recorded and analysed using outputs from ©ATracker and Python on Microsoft Excel.

**Result:**

Three of the residents spent the majority of their waking hours in a neutral state, showing minimal indicators of sensory stimulation, social engagement, or supervised independence. Self‐care practices were low or absent for all but one resident. While inappropriate or antisocial behaviours were rarely observed, the lack of meaningful engagement and proactive care highlights significant gaps in dementia care practices.

**Conclusion:**

This study lays the groundwork for designing tailored interventions aimed at enhancing current dementia care practices by providing person‐centred dementia care and effectively responding to the needs and preferences of people living with dementia.